# Improvement of Surface Properties of Carbon Steel Through Electrospark Coatings from Multicomponent Hard Alloys

**DOI:** 10.3390/ma18102211

**Published:** 2025-05-10

**Authors:** Todor Penyashki, Georgi Kostadinov, Mara Kandeva

**Affiliations:** 1 Institute of Soil Science Agrotechnologies and Plant Protection “N. Pushkarov”, Agricultural Academy, Shose Bankya Str. 7, 1331 Sofia, Bulgaria; gdkostadinov@mail.bg; 2 Faculty of Industrial Technology, Technical University of Sofia, Kliment Ochridsky 8, 1000 Sofia, Bulgaria; kandevam@gmail.com

**Keywords:** electrospark deposition (ESD), phase composition, coatings, roughness, microhardness, carbides, nitrides, borides, wear resistance

## Abstract

This work demonstrates the possibility of creating effective composite coatings with a complex structure and phase composition on carbon steel C45 via electrospark deposition (ESD) with multicomponent electrodes with a bonding mass composition of Co-Ni-Cr-B-Si semi-self-fluxing alloys and superhard compounds WC, B_4_C and TiB_2_. The variation in the roughness, thickness, composition, structure, microhardness and wear at the friction of the coatings as a function of the ratios between the bonding mass and the high-hardness components in the composition of the electrode and of the pulse energy for ESD has been studied. It has been established that with a content of the bonding mass in the electrode of 25–35%, coatings with improved adhesion and simultaneously higher hardness and toughness are obtained. Suitable electrode compositions and optimal pulse energy have been defined, which provide dense and uniform coatings with an increased amount of crystalline-amorphous structures, as well as intermetallic and wear-resistant phases, with thickness, roughness and microhardness that can be changed by the ESD modes in the ranges of δ = 8–65 µm, Ra = 1.5–7 µm, and HV 8.5–15.0 GPa, respectively, and minimal wear of the coated surfaces that is up to 5 times lower than that of the substrate and up to 1.5 times lower than that obtained with conventional WC-Co electrodes.

## 1. Introduction

Wear-resistant coatings are a technically and economically viable way to strengthen and regenerate the surfaces of machine elements subjected to intense wear. The numerous operating and friction conditions under which wear occurs have led to the creation of numerous technologies and methods for applying coatings, such as electrochemical, galvanic, thermochemical, vacuum, electrophysical, sol–gel methods and many others. However, the applications of most of these technologies are often associated with a number of technological difficulties and limitations due to reasons such as expensive and energy-intensive equipment, complex technologies requiring significant investments, the heating of the parts leading to recrystallization and thermal deformation, insufficient adhesion and thickness of coatings, the need for subsequent surface treatment, inaptitude for strengthening a wide range of working elements with different masses and configurations, high costs of the deposition process, as well as some shortcomings of the coatings, environmental pollution, etc. For example, wear-resistant coatings obtained by the popular and established physical and chemical deposition from vapor phase methods (PVD and CVD) have high hardness and wear resistance, but they require expensive equipment and infrastructure. In addition, they cannot provide coating thicknesses above 30 µm, good adhesion and application on complex surfaces. Laser coating tools and equipment are complex and expensive, and it is difficult to achieve a coating on a product with complex geometry. The ion–plasma nitriding process takes 3–6 h, and the formation of cathodic arcs damages the surface during processing. Flame spraying requires pre-texturing and heating and post-treatment of the coated surfaces. Some of the methods are too expensive and can only be used for applications where the high cost is justified.

The above difficulties and limitations can be overcome by using the electrospark deposition (ESD) method, which is much cheaper and easier than the other methods and does not require large investments and additional infrastructure, and its versatility, simplicity, flexibility and high efficiency allow its successful use for obtaining wear-resistant coatings on any metal parts, tools and parts in all industrial sectors [1,2,3]. The most significant advantages of ESD over other methods are low energy consumption and cost; high adhesion strength to the substrate; the absence of heating and deformation of the coated product; the simplest, easiest and most universal technology; the possibility of local application of coatings on any surfaces, even those with the most complex shape; the possibility of operation without additional treatment of the modified surfaces; and the high environmental friendliness [2,4,5].

The main disadvantage of the method is the relatively low productivity, which makes it less suitable for large series of parts compared to metallization methods, electrochemical methods and PVD. Another disadvantage is the inability to obtain high-quality coatings with increased thickness. Increasing the thickness of the applied coating is associated with an increase in its roughness and surface defects, which negatively affects its operational properties and leads to the need for subsequent processing to reduce the roughness. Due to the choice of electrode materials and process parameters that are unsuitable for specific products and their operating conditions, the results are not always sufficiently satisfactory.

ESD is based on the use of pulsed discharges with controlled energy and a duration of 10^−6^–10^−5^ s. As a result, electrical erosion and the polar transfer of material from the deposition electrode (anode) to the product (cathode) occur. According to the works of [5,6,7,8], the pressure of the shock wave from the action of the electric spark is from 2 × 10^6^ to 7 × 10^6^ MPa, and the temperature of the electrode surface reaches 5 × 10^3^ to 1 × 10^4^ °C. The intensity of the electric field during ESD reported by researchers in [2,5,6,7,8,9] is 10^7^–10^9^ W/cm^2^, which is much higher than the critical intensity at which the anode material melts. As a result, the explosively molten electrode material is transferred onto the cathode surface in liquid, solid (softened) and vapor phases with extremely high particle velocity. It mixes with the locally melted micro-spot of the substrate, and a surface layer of a mixture of the two materials is formed, such as new compounds and phases resulting from their chemical interaction and from reactions with the environmental elements. According to many researchers [1,2,5,6,8,9,10,11,12], the short duration of the ESD process and the extremely high heating rates up to 10^4^ °C and cooling rates (up to 10^5^–10^6^ °C/s) allow for the formation of new ultradispersed, amorphous and nanoscale wear-resistant structures, as seen in many works [1,2,5,6,7,9,10,11,13,14,15,16,17]. For example, in their works, Liu et al. [9], Hasanabadi et al. [10], Petrzhik et al. [13] and Cadney et al. [14] demonstrated the possibility of using ESD to obtain a surface layer with an amorphous–crystalline structure, as well as to form amorphous iron-based metal layers with improved properties on steel substrates by using both amorphous and crystalline electrode materials. After performing ESD on a high-speed steel substrate, Xiang Wei et al. [12] obtained a dense, metallurgical material bonded to the substrate cermet coating based on Mo_2_FeB_2_, with an average thickness of 48.1 μm, a microhardness of 1275 HV200, a lower friction coefficient and better wear resistance compared to the high-speed steel substrate. In the ESD process, Wei transformed the cermet into a martensite phase, which has a nanocrystalline structure (31.7 nm), and an amorphous phase.

The heat-affected zone is small, resulting in minimal thermal and metallurgical changes. The layer has increased adhesion to the substrate, as it is formed by mixing the molten materials of the processing electrode (anode) and the substrate (cathode), resulting in increased hardness and tribological and corrosion properties, which are controlled in a wide range by changing the parameters of the spark discharge and the composition of the electrode material.

For electrodes in ESD, a wide range of metals and metal alloys [1,2,3,5,8,9,10,14,16,17,18,19,20] and classic hard alloys based mainly on WC-Co, WC+TiC–Co, etc., [1,2,3,4,5,12,15,19,21,22,23,24,25,26,27] are usually used. As shown in the works of [1,2,3,4,5,8,9,10,13,14], metals and alloys have high strength and ductility and provide good adhesion to the base and higher thickness and density of the coating but have limited hardness and wear resistance. The hard alloy coatings according to [1,2,3,4,5,6,15,19,21,22,23,24,25,26,27] have high hardness and wear resistance, as well as high brittleness, which does not allow for the use of high-energy pulses due to the deterioration of their quality and in many cases limits the desired effect in a number of applications.

It is obvious that further enhancement of the ESD effect is associated with obtaining coatings from harder and more wear-resistant materials that provide an increase in thickness, adhesion and uniformity, as well as hardness, elasticity, strength and wear resistance of the obtained coatings. The data from numerous studies [1,2,5,15,17,19,25] show that such a complex balance of contradictory properties can be achieved using composite multicomponent and multiphase coatings consisting of several highly wear-resistant materials and a solder mass from several metals or metal alloys. Tarelnik and his colleagues [28] managed to obtain a multicomponent quasi-multilayer wear-resistant coating on steel with increased thickness, a roughness of Ra 7.7 μm and a microhardness of 11.5 GPa.

Multicomponent coatings are a modern trend in new technologies, which are rapidly developing and expanding, and are the subject of numerous studies [1,2,5,12,17,18,23,25,28,29,30,31,32].

Paustovskii and colleagues [30] reported the development of electrode materials based on (Ni-Ni_3_B-Cu-Si) with additives of titanium carbide, chromium carbide and tungsten carbide, which allow for the production of coatings with a thickness of up to several mm. They also developed alloys based on B_4_C-TiB_2_. With these alloys, a protective coating with a thickness of up to 100 μm with high hardness (32–43 GPa) and wear resistance was formed. Mihailov et al. [31] obtained multicomponent coatings using a modernized version of ESD with the introduction of an additional powder material into the interelectrode gap. Coatings with numerous double and triple wear-resistant phases, an amorphous-crystalline structure with a hardness of up to 15 GPa and wear up to six times lower than that of steel substrates were obtained.

The literature data [3,5,6,22,25,29,30,32,33,34,35,36] and accumulated experience show that the productivity of the ESD process and the transfer from the liquid phase can be increased, and the brittle destruction of the electrode in the spark discharge process is reduced by increasing the amount of metal-binding mass in the composition of the hard alloy material and by using a binding mass of components that form high solubility or intermetallics with the steel substrates. In addition, the increased amount of metal binders will better wet the refractory phases of the composite, thereby providing good adhesion to the alloyed surface. On the other hand, their reduced amount increases brittleness and reduces adhesion and resistance to high pressure, which can lead to the destruction of the coatings.

In this context, the study of ESD coatings from multicomponent hard alloy electrodes with different ratios between their constituent components, which imply the formation of a predominant liquid phase in which the components of the electrode and the substrate are actively mixed and spread evenly over the coated surface, is of significant interest to science and practice.

The purpose of this work is to investigate the influence of the ratios between the components in the composition of multicomponent electrode materials on the quality of the coatings with the goal of obtaining coatings with improved characteristics, properties and wear resistance.

## 2. Materials and Methods

### 2.1. Selection of the Coating Material

The main requirements for ESD coatings on steel surfaces have been indicated in our previous studies [29,32,34] and can be reduced to the following: high wear resistance and the preservation of consumer properties in the entire range of changes in temperature, pressure and chemical aggressiveness in the working area; high microhardness, which represents the resistance of the coating to abrasive wear; strength and toughness, which reflect the ability of the coating to withstand stresses and deformations in working conditions and to prevent the formation and development of cracks under conditions of variable loads, shocks and vibrations; chemical resistance; high-quality characteristics, such as low roughness, high density and uniformity; the necessary thickness for the specific case; a low coefficient of friction; and good compatibility with the base material.

The above-mentioned requirements are met to the greatest extent mainly by metals from the IV–VI groups and from the VIII group from the periodic table (Fe, Co, Cr, Ni and Ti,) and their highly hard and wear-resistant compounds, such as carbides, carbonitrides, nitrides and borides.

There are a lot of data in the literature on the electrode materials used and their influence on the topography, morphology, composition, structure and properties of the coatings deposited with them [1,2,3,4,5,8,9,10,11,12,14,15,16,18,19,20,21,22,23,24,25,26,29,30,31,32,33,34,35]. Based on the analysis of the data and the results of our previous studies [22,29,32,34,36], the following composition was chosen for the multicomponent coating electrodes for the ESD process: WC-TiB_2_-B_4_C-Co-Cr-Ni-B-Si-Fe-C. Mixing materials with high hardness and wear resistance (carbides and borides) with metals with a lower melting point allows for the production of coatings with favorable integration, the most complete use of the advantages and properties of each of the individual components and obtaining additional fundamentally new components and new favorable properties as a result of chemical reactions and interactions in the process of plasma-spark discharges. The lower-melting-point components will form a fine-grained metal matrix that is firmly bonded to the base and saturated with fine particles of high hardness and wear resistance while simultaneously possessing high strength and high wear resistance. In addition to high wear resistance, the metal matrix will act as a friction lubricant to prevent the formation of wear craters and the welding of the rubbing surfaces.

As a basis for the binding mass, ready-made powders from the commercial network, manufactured by the company “Castolin” under the brand name Eutaloy10611, were used, in which some slight adjustments were introduced in order to adapt them to the specifics of the ESD process. The chemical composition of the binding mass, conditionally designated by us as KW, is given in Table 1.

Due to its good wear resistance and solubility in iron, the low-melting semi-self-fluxing NiCrBSi alloy is widely used to obtain coatings on steels, as works [37,38,39,40,41,42,43,44] report a significant increase in the wear resistance of coated products. The elements B and Si in this composition reduce the melting point of Ni and form a low-melting borosilicate, which flows to the surface and protects the melt from oxidation. The low melting point of NiCrBSi alloys, as well as the fluxing effect of Si and B, allows these materials to be easily deposited by ESD, as evidenced by the data presented in the works of [29,30,32,33,34,35].

The beneficial influence of Co, Ni and Cr on the wear resistance of the coated surfaces is reflected in the studies of [3,4,5,6,15,25,28,29,30,33,35,36,40,41,42,43]. It has been shown that carbon and boron can serve as donors for the formation of new wear-resistant carbides and borides in the process of spark-plasma discharges. Co, Ni and Cr are suitable for improving the transfer and adhesion with steel substrates, as well as for forming a strong matrix for fixing the carbide and boride particles in the layer and obtaining dense and uniform coatings with higher thickness and wear resistance.

For wear-resistant components in the composition of the electrodes, a mixture of popular and proven effective coating materials was selected, namely the most famous and widely studied representative of the metal–ceramic coatings, WC-8Co, the interest in which continues to this day [1,2,3,4,5,19,20,21,22,23,24,25,26,27,41,42,43,44], as well as high-hardness B_4_C used for ESD coatings by the researchers [15,30,32,33,35] and TiB_2_, the coatings of which were studied in the works of [30,32,33,34,35,45,46,47,48]. It is widely known that the surfaces coated with these compounds have much higher wear resistance than that of the base metal.

Tarelnik et al. [25] reported on obtained coatings from sintered electrode materials containing 10–30 wt.% of the (Ni–Cr–Si–B) alloy WC, with a thickness of up to 100 μm and a microhardness of 12.3–14.2 GPa. The wear resistance and service life of these coatings are significantly higher than those made from standard hard metal WC.

The use of multicomponent electrodes from NiCrBSi–WC and the formation of coatings with increased hardness and wear resistance compared to those of coatings from NiCrBSi alloys are reported in the works of [35,41,42,43,44]. The developed materials are promising due to their application as electrodes in the ESD of structural steels and titanium alloys. Umansky et al. [45,48] reported the creation of coatings from the TiB_2_-(Fe-Mo) system and NiCrBSi–20 wt.% TiB_2_ with increased hardness and wear resistance. It was found that by increasing the amount of metal phase in the electrode material, the manufacturability of the electrospark alloying process improves, but the wear resistance of the resulting coatings under abrasive wear conditions decreases. The optimal amount of the metal phase was determined.

Many researchers [3,4,5,13,14,15,29,30,33,35,41] recommend an “optimal” amount of the solder mass of 12–25% for coatings with friction purpose and 20–40% for coatings with increased thickness and toughness. Based on their statements, several different experimental ratios between the metal and ceramic components are selected and given in Table 2, which suggest possibilities for creating simultaneously high wear resistance and strength, as well as for obtaining new additional wear-resistant compounds and phases in the process of forming the coatings.

The selected electrode compositions combine the advantages of the above-described bonding and high-hardness materials and differ from the known ones in that they simultaneously contain highly wear-resistant WC, TiB_2_ and B_4_C, and the binder mass built from the multicomponent Ni-Cr-Co-B-Si-C-Fe system can provide higher transfer, higher coating density and a strong bond with the base. In addition, with these compositions, it is possible to form amorphous and nanoscale structures, which, according to many authors [1,2,5,6,8,9,10,11,13,14,15,16,17,39], provide a stronger bond with the substrate, stronger bonds between the particles in the coating and improved wear resistance. Coatings made of the classic hard alloy electrode material WC-Co8 with the designation WK8, which was previously studied and investigated, were used as a reference for comparison.

The ESD electrodes were made using the following powder metallurgy methods: joint dry mixing and grinding of the mixtures to a particle size of 10–20 µm, homogenization and plasticization, pressing in the form of prismatic samples (6 × 4 × 40 mm) and sintering in a vacuum furnace in a protective inert environment of hydrogen.

### 2.2. Materials and Equipment for ESD and Deposition Conditions

- *Substrate*

Model plates of the widely used carbon steel C45 (0.45% C, 0.4% Si, 0.6% Mn, 0.02% P and 0.03% S), with Fe being the rest) with a hardness of 190–210 HB, sizes of 10 × 10 × 4 and 12 × 12 × 4 mm and roughness values of Ra ≈ 1 μm and ≈2.5 μm are used as the substrate.

- *Equipment for ESD*

In order to reduce the size of the erosion craters on the substrate surface and obtain a smoother and more uniformly coated surface with minimal surface defects, a low-energy apparatus with a vibrating electrode “Hardedge” from Great Britain was used for ESD with the following parameters:

- Short-circuit current—0.1–2 A;
- Voltage—U = 80 V;
- Capacitance—C = 5–100 µF;
- Energy of a single pulse—E = CU2/2; E = 0.02–0.3 J;
- Vibration frequency—100 Hz;
- Pulse duration ≈ 100 µs.

Deposition was performed in atmospheric conditions with the application of three consecutive deposition runs one on top of the other at an average productivity of ≈10 min/cm^2^ for the three deposition runs. Processing modes are given in Table 3.

### 2.3. Types of Research, Methodology of Measurements and Research Equipment

The roughness parameters of the coatings (average roughness—Ra; root mean square roughness—Rq; maximum profile height—Rt; the average value of the 5 highest protrusions and 5 deepest depressions of the profile within the basic length—Rz) were measured with the profilometer “AR-132B” (Shenzhen Graigar Technology, Co., Ltd., Shenzhen, China) according to the ISO 21920:2021 standard [49] in two mutually perpendicular directions in five sections. The arithmetic average values, standard deviation and confidence interval were determined. The sharply different values were rejected using the Grubbs method.

The thickness δ was measured with a dial indicator with an accuracy of 0.001 mm. The results are the arithmetic mean of 5 parallel measurements.

Vickers microhardness (HV) was measured from above (on the top of the coating) after smoothing the surface irregularities. The hardness tester “Zwick 4350”-(Zwick Roell, GmbH & Co., KG, Ulm, Germany) equipped with a Vickers diamond prism indenter at a load of 0.2 N (20 g) for a time of 10 s was used. In order to eliminate the influence of the substrate, the measured hardness was calculated according to the methodology presented in [50]. The number of parallel measurements was 10.

Microstructural, topographic and morphological analyses of the coatings were performed with the metallographic optical microscope “Neophot 22” (Carl-Zeiss, Jena, Germany) and the scanning electron microscope (SEM-EDS) “EVO MA 10 Carl Zeiss” with a built-in X-ray microanalyzer EDX system from “Bruker”.

The phase composition was investigated with a Bruker D8 Advance X-ray diffractometer (Bruker AXS, Karlsruhe, Germany) in “Cu Kά” radiation.

The distribution of elements in the surface layer was investigated via X-ray using Energy-Dispersive Spectroscopy (EDS) with an X-ray energy dispersion microanalyzer EDX system from “Bruker”, the scanning electron microscope SEM “EVO MA10 Carl Zeiss” (Carl Zeiss Microscopy GmbH, Germany) and an X-ray energy-dispersive microanalyzer EDX system from “Bruker” (Bruker AXS, Karlsruhe, Germany).

Scratch tests and the determination of the coefficient of friction (μ) and the tangential force (Ft) were performed with a CSM REVETEST Scratch Macrotester (Anton Paar GmbH-Ostfildern-Scharnhausen, Germany) with a Rockwell C diamond indenter with a tip radius of 200 μm in progressive loading mode with a normal force range from 0 to 50 N at a speed of 10 N/mm. Light optical microscopy of the scratch marks was performed using a Nikon MA200 microscope from Tokyo, Japan.

Tribological tests were carried out with a “Finger-Disc”-type tribotester under dry friction conditions with firmly fixed abrasive particles (corundum abrasive paper with a P320 grain size) in planar contact between the coated samples with dimensions 12 × 12 × 4 mm and the abrasive paper at a load of 5 and 10 N, respectively, a nominal contact area of 1.44 cm^2^, nominal contact pressure values of 3.47 and 6.94 N/cm^2^ and a sliding speed of 0.238 m/s. Mass loss (mass wear) was determined as the difference between the initial mass of the sample “m0” and its mass “mi” after a certain sliding distance, where m = m0 − mi, reported in mg. The wear rate was defined as the amount of wear per unit friction path: I = m/L, mg/m, where m is the mass wear for the friction path L under normal load P. Wear resistance was defined as the reciprocal of the wear rate. The results are the arithmetic mean of 3 parallel experiments. The mass of the samples before and after a given friction path was measured with the electronic balance WPS 180/C/2 (RADWAG, Radom, Poland) with an accuracy of 0.1 mg.

## 3. Results and Discussion

### 3.1. Coating Characterization—Roughness, Thickness and the Topography and Structure

**Figure 1:**
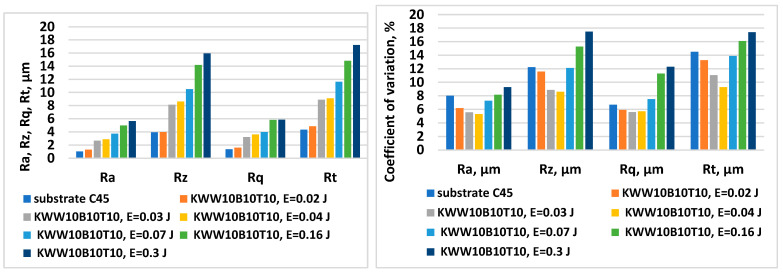
Variation in the roughness parameters of the electrode coated with KWW10B10T10 as a function of the pulse energy.

Figure 1 shows the change in the roughness parameters and the coefficient of variation in the coatings from the KWW10B10T10 electrode as a function of the pulse energy.

**Figure 2:**
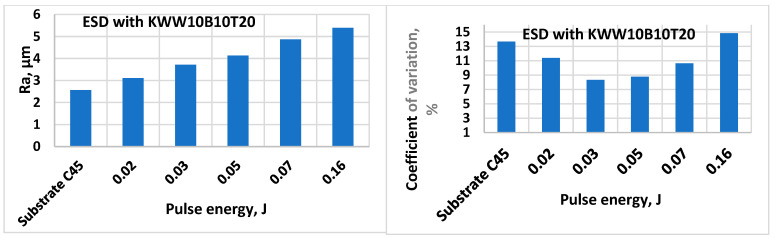
Variation in the roughness Ra of the electrode coated with KWW10B10T20 as a function of the pulse energy.

Figure 2 shows the variation in the roughness Ra and the coefficient of variation in the electrode coated with KWW10B10T20 as a function of the pulse energy, which is analogous to that in Figure 1.

The results obtained show that the increase in pulse energy (in the direction from mode No. 1 to mode No. 6—Table 3) leads to an increase in the roughness parameters of the obtained coatings, but their unevenness also increases significantly. This is characteristic of the ESD process and is explained by the increasing portions of the molten and transferred electrode material onto the cathode as well as the increasing sizes of the cathode micro-areas melted by spark discharges. It is noticeable that for both electrodes presented in Figure 1 and Figure 2, the standard deviation in ESD is lower than that of the mechanically treated surfaces of the substrates, and with the increase in the pulse energy to E = 0.03–0.05 J, the deviation decrease, reaching the minimum value, after which the deviation gradually increases.

Similar relationships were obtained with the other selected electrode compositions from Table 2. Table 4 shows the range of variation in the roughness parameter Ra, thickness δ, microhardness Hv and the strengthening coefficient of the coatings deposited with the selected electrodes at the pulse energy in the range of E = 0.03–0.3 J. Table 5 shows the average values of the roughness parameters, the minimum and maximum measured value of the thickness of the coatings δ and cathode growth during ESD with KWW electrodes with the maximum and minimum amounts of the metal matrix (40 and 22%, respectively) at pulse energy values of 0.05 and 0.07 J.

The results show that at the same pulse energy, the roughness parameters and the thickness of the coatings are different for the different electrodes studied. In the modes with a pulse energy of 0.02 J, the values of the roughness parameters are the lowest and are comparable to those of the substrate (Figure 1 and Figure 2), with the coatings from the different electrodes showing close values of the roughness parameters. With an increase in the pulse energy to 0.03 and 0.05 J (Figure 1, Figure 2 and Table 4 and Table 5), the parameter Ra increases to values of Ra ≈ 3–4 µm, with the differences in the values of the roughness parameters of the coatings from the different electrode compositions also increasing. In parallel with the roughness, the thickness of the coatings also increases. The differences in the roughness parameter Ra at the maximum and minimum ratios between the solid phases and the metal bond are on the order of 20%, and those for the thickness of coatings are about 25%.

The maximum thickness of the coatings on the order of 60–65 μm at a roughness of Ra ≈ 5 μm was registered in ESD with the electrodes with the highest content of the brazing metal mass (KWT10, KWB10T10 and KWW10B10T10) in the mode with the maximum used pulse energy of E = 0.3 J. At the same energy, the thickness of the coatings applied with the WK8 electrode is 45 μm.

The following differences are observed in the coatings from the studied electrode compositions:

- In the coating with a minimum content of the solder mass (KWW10B20T20), at low pulse energies up to 0.03 J, the lowest roughness on the order of Ra ≈ 2–2.5 µm was recorded, and with an increase in energy above 0.07 J, the roughness increases almost abruptly; at the maximum energy of E = 0.3 J, it reaches Ra ≈ 7 µm. In the coatings from these electrodes, at E ≥ 0.05 J, the amount of material transferred from the brittle fracture of the electrode increases, as well as the unevenness and structural defects of the coatings, and accordingly, the roughness parameters also increase, and the gradient of thickness increase decreases.
- For coatings with a maximum content of binding mass of 40%, when electrode KWT10 is at low energies of E = 0.02 and 0.03 J, the roughness of the coatings is comparable to, or higher than, that of KWW10B20T20 electrodes, but at E ≥ 0.05 J, the roughness parameters show lower values, and the thickness shows higher values than those of coatings from KWW10B20T20 and from the other electrodes. Therefore, the use of electrodes with a higher content of the solder metal mass makes it possible to obtain coatings with increased thickness at lower roughness.

**Figure 3:**
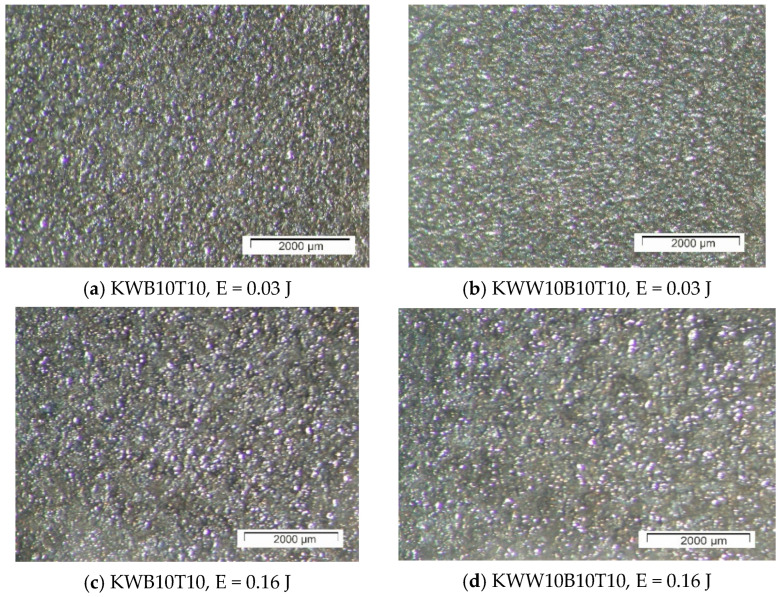
Photographs of the topography and surface relief of the KWW10B10T10 and KWB10T10 coatings deposited on C45 steel at low and high pulse energies.

**Figure 4:**
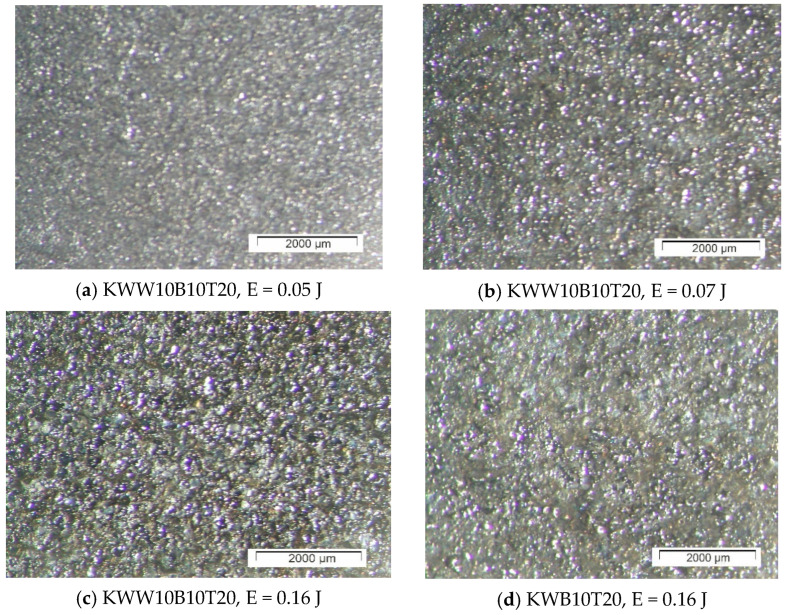
Photographs of the topography and surface relief of the KWW10B10T20 and KWB10T20 coatings deposited on C45 steel at low and high pulse energies.

**Figure 5:**
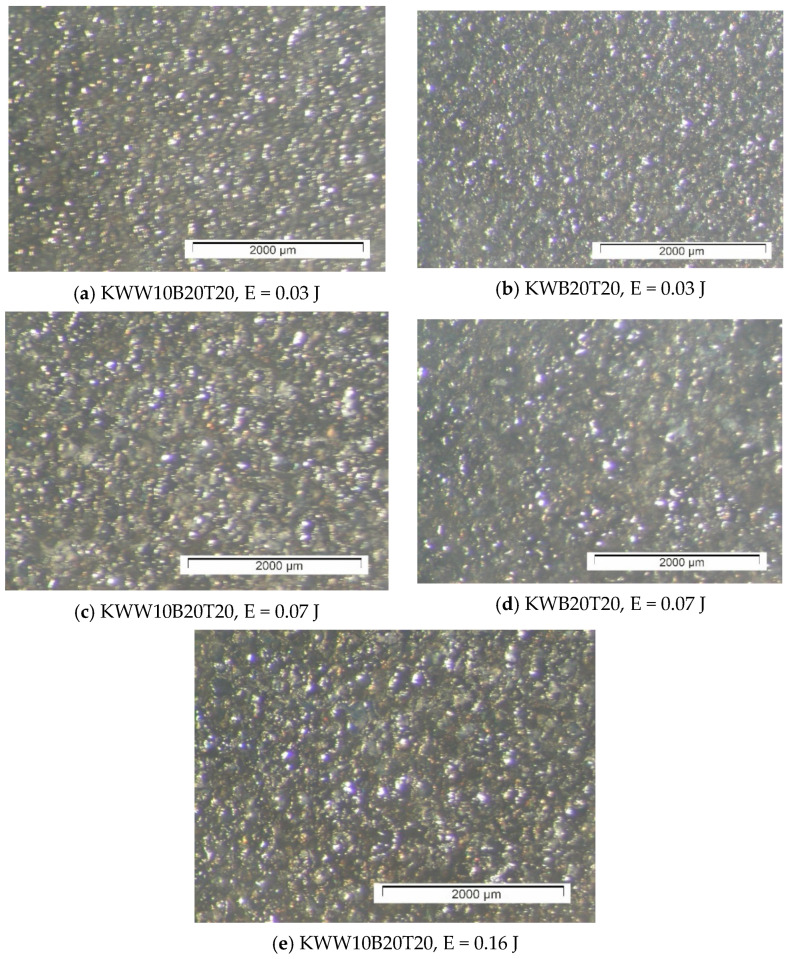
Photographs of the topography and surface relief of the KWW10B20T20 and KWB20T20 coatings deposited on C45 steel at low and high pulse energies.

**Figure 6:**
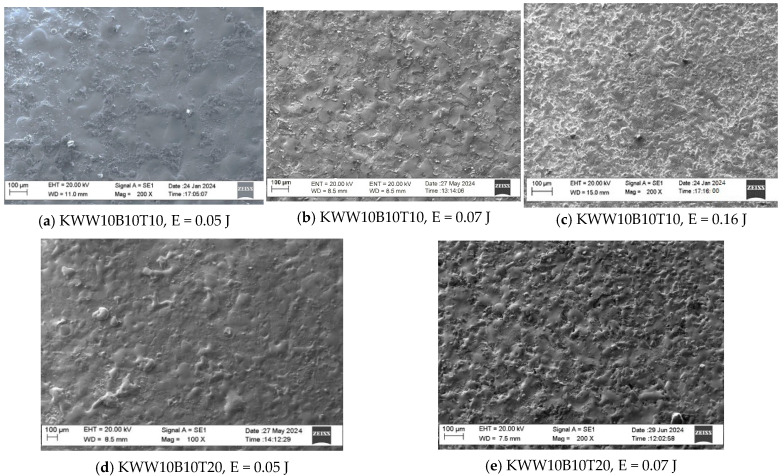
SEM images of the topography and surface relief of coatings from the KWW10B10T10 and KWW10B10T20 electrodes deposited on steel 45 at different pulse energies.

**Figure 7:**
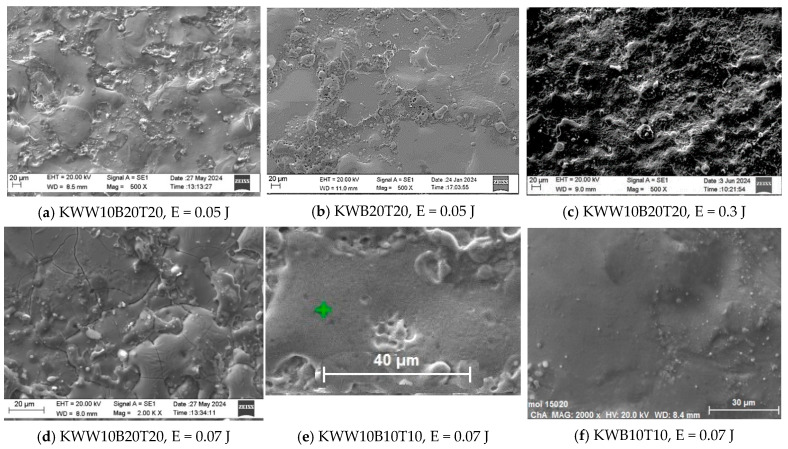
SEM images of the topography and surface relief at different magnifications of coatings from the studied electrodes deposited on steel 45.

Figure 3, Figure 4 and Figure 5 show typical photographs of the topography and surface relief of coatings from the studied electrode compositions obtained with an optical microscope, and Figure 6 and Figure 7 show typical SEM images at different magnifications. From the presented figures, it can be seen that dense and similar in structure coatings were obtained on the steel surfaces, with a specific relief different from the original, and these coatings were formed mainly from a liquid phase. The areas formed from the liquid phase are clearly visible in Figure 6 and Figure 7. The coatings obtained with the studied electrode materials under different regimes differ mainly in thickness and uniformity.

SEM observations of the surface relief (Figure 6 and Figure 7) show the presence of microscopic irregularities at different places on the surface of the coatings. The electroerosion craters and the smooth surface areas between them are clearly distinguished. Three zones are clearly visible on the samples, namely convex areas (bumps); smooth, glass-like zones; and zones formed by small particles with slight irregularities. The comparison of the ESD surfaces shows that the coatings obtained from all the studied electrode compositions are most uniform at E ≤ 0.03 J (Figure 3a,b, Figure 5a,b and Figure 6a,d). With increasing pulse energy, the sizes of the individual structural components increase, and at E ≥ 0.05 J, individual irregularities and accumulations appear, the amount of which increases with a further increase in pulse energy (Figure 4, Figure 5, Figure 6a–c and Figure 7a,b,d). At these energies, intense heating of the electrodes occurs, contributing to predominant solid-phase erosion and the appearance of protrusions.

The most uniform is the surface of the coatings from the electrodes with 10% TiB_2_ and B_4_C content, namely KWB10T10 and KWW10B10T10 (Figure 3, Figure 6a,b and Figure 7e), which retain their density and uniformity at energies up to 0.07 J (Figure 3, Figure 6a,b and Figure 7d). The coatings obtained with these electrodes have smaller structural components and a smaller number of cracks and pores caused by the solid-phase transfer of the resulting higher pulse energy. In the coatings from the electrodes with a higher content of solid phases (Figure 4, Figure 5, Figure 6d,e and Figure 7a–d), a larger number of convex areas, microcracks and pores are observed, which are most pronounced in the electrodes with the highest content of high-hardness phases (Figure 7c,d).

Increasing the amount of B_4_C and TiB_2_ to 20% in the KWW10B20T20 and KWB20T20 electrodes led to the appearance of individual irregularities and single accumulations at E = 0.05 J (Figure 5, Figure 6d,e and Figure 7a–d), which continue to increase with a subsequent increase in energy (Figure 5 and Figure 7c,d). The highest degree of unevenness and roughness is observed at high pulse energy values of E = 0.07 (Figure 4), 0.16 (Figure 5e) and 0.3 J (Figure 6 and Figure 7c). Apparently, the 20% content of TiB_2_ and B_4_C reduces the strength of the electrode, as the transition from brittle fracture begins at a lower energy level, and the coatings become visibly more uneven with higher roughness than those of the electrodes with the 10% content of these two compounds. Therefore, in order to obtain a dense layer without microcracks, it is necessary to maintain the energy level below certain values, which are different for each specific electrode.

Results on the influence of the ratios between the solid phase and the metal phase in the current electrode materials have been presented in the works of a number of researchers [1,3,4,5,15,23,25,30,35,41,44,45,46,47,48], who assert that increasing the amount of the metal phase in the electrode allows for the production of more uniform coatings with lower roughness and increased thickness. The electrode compositions used by us allow for the formation of coatings with lower roughness and a thickness comparable and even higher than that reported by the above authors at lower pulse energy levels.

From the images in Figure 3 and Figure 4 and those in Figure 5, Figure 6 and Figure 7, it can be concluded that the content of B_4_C has a stronger influence than that of TiB_2_ on the homogeneity and quality of the coatings. The comparison of the coatings from the “KW” and “KWW” electrodes (Figure 3, Figure 4c,d, Figure 5a–d and Figure 7) shows that the surface of the two types of coatings is similar, but the relief of the “KW” coatings is smoother and more uniform.

In the SEM images, smooth, glass-like areas obtained from the mixing of the molten electrode material and the molten cathode spot result in lighter particles with an irregular shape (Figure 6a,b and Figure 7a,b), which are clearly unmelted carbides and borides, and “splashes” of small particles poorly attached to the molten metal matrix on the surface of the coatings (Figure 6a,b and Figure 7a,b,e) are distinguished. At a higher magnification in the coatings from these electrodes deposited with a pulse energy of E ≥ 0.05 J, in addition to the uneven relief, individual pores and microcracks are also observed (Figure 7a,d), the amount of which increases with increasing pulse energy.

**Figure 8:**
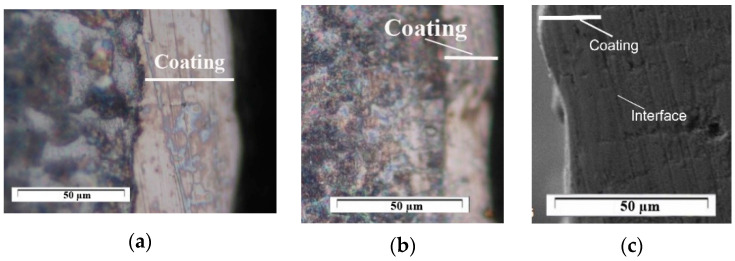
Cross-section of coatings from the KWW10B10T10 (**a**), KWW10B10T20 (**b**) and KWW10B20T20 (**c**) electrodes deposited at a pulse energy of E = 0.07 J.

Figure 8 shows the cross-section of coatings from the KWW10B10T10, KWW10B10T20 and KWW10B20T20 electrodes deposited at a pulse energy of E = 0.07 J.

As can be seen from Figure 8, the coatings have a compact and uniform microstructure. The study of the microstructure of the coatings obtained with the studied electrodes showed that by increasing the amount of the brazing metal phase, their uniformity and thickness increase. On the other hand, however, it was found that by increasing the pulse energy above certain limits so that it is different for each specific electrode composition, the fraction of the transferred material from brittle fracture increases sharply, which worsens the quality, uniformity and roughness of the obtained coatings. In addition, increased pulse energy leads to other negative effects such as overheating of the electrode, the appearance of a thermally affected sublayer and the appearance of cracks and defects in the coatings, so in order to avoid the aforementioned defects, the energy should be increased up to certain limits that are specific for each electrode material.

The main reasons for the lower thickness of the coatings of the KWWB20T20 and KWW10B20T20 electrodes can be indicated as the heating of the electrode and the subsequent brittle chipping and transfer of unmelted particles onto the cathode. Moreover, as can be seen from Figure 1 and Figure 2 and Table 5 and Table 6, the higher the amount of the binder in the composition, the higher the threshold of brittle fracture of the electrode and the coating; i.e., increasing the amount of binding metals in the composition of the electrode material allows us to create a coating with a greater thickness, as required by practical needs.

The summary of the results of the measurements (Figure 1 and Figure 2 and Table 5 and Table 6) and microscopic observations (Figure 3, Figure 4, Figure 5, Figure 6, Figure 7 and Figure 8) allows us to conclude that the coatings from KWT10, KWB10T10 and KWW10B10T10 are more uniform and have better geometric characteristics and structure. The maximum thickness at which relatively uniform coatings with roughness up to Ra = 3–4 µm are obtained by ESD at E = 0.07 J is on the order of 45–50 µm. At the same pulse energy, the thickness of the coatings from the conventional electrode WK8 reaches 35 µm. The higher thickness of the “KW” coatings than that of WK8 can be explained by the presence of a greater number and amount of binding metals forming unlimited solid solutions with iron, as well as the presence of boron and silicon in the alloying electrode, which slow down the formation of oxide films, have a positive effect on the continuity, and increase the thickness of the coating. In addition, the presence of boron and boron carbide reduces the erosion resistance of the electrode [22,35,39,41,45,46,47,48], thus increasing the transfer of the electrode material to the treated surface.

### 3.2. Phase Composition of Coatings

**Figure 9:**
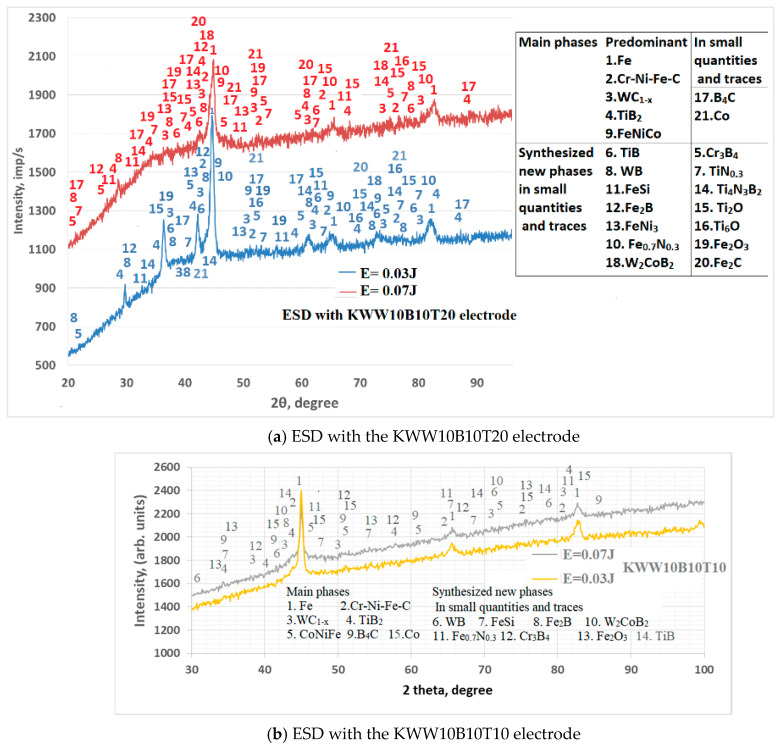
XRD diffraction patterns of coatings from electrodes at different pulse energies.

**Figure 10:**
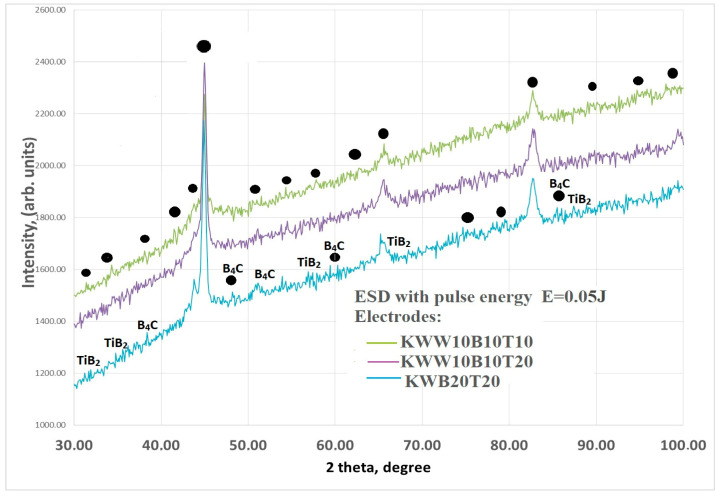
XRD diffraction patterns of coatings from electrodes at a pulse energy of 0.05 J.

Figure 9 shows the X-ray diffraction patterns of coatings from the KWW10B10T20 electrode applied at pulse energies of E = 0.03 and 0.07 J (a), as well as patterns of coatings from KWW10B10T10. Figure 10 shows the X-ray diffraction patterns of coatings at a pulse energy of 0.05 J.

The X-ray diffraction patterns of the coatings from the other selected electrodes are similar (Figure 10). Since several different phases correspond to each of the registered characteristic peaks at a specific angle of 2θ, all available phases are noted in the X-ray diffraction patterns in Figure 9, the presence of which is registered at least at three diffraction peaks. The corresponding diffraction angle of 2θ of the registered phases is given in Table 6. The X-ray diffraction patterns of the coatings from the studied electrode compositions are similar, and their phase composition is close. The main differences are in the intensity and width of the characteristic peaks of the phases.

Increasing the pulse energy causes an increase in the amount of transferred material, an increase in the degree of partial decomposition of WC, TiB_2_ and B_4_C and, accordingly, an increase in the amount of high-hardness and newly formed phases and the degree of alloying of the layer. Due to the dissociation of carbides and borides from the electrode materials, new intermetallic, carbide and boride phases appear, the amount of which increases with increasing pulse energy. At values above 0.05 J, traces and small amounts of iron and titanium oxides appear in the composition of the coatings, which increase with a further increase in energy. The structural maxima also expand, which are more pronounced in the coatings from the electrodes with the highest amount of bonder metal mass, such as KWT10, KWB10T10 and KWW10B10T10.

As can be seen from Figure 9 and Figure 10, a complex multicomponent matrix of Fe, Co, alloyed austenite, such as Cr-Ni-Fe-C or Cr-Ni-Fe-Co-C, and intermetallic compounds, such as FeNiCo, FeNi_3_, Co_7_Fe_3_ and Fe-Ni, with embedded finely dispersed carbides, nitrides and borides from the electrode material, as well as new ones obtained during the reaction of the elements from the electrode, the substrate and the surrounding air environment, is registered in the coatings. W_2_C and WC_1−x_ with small inclusions of WC are registered in the coatings, but traces of WB, WN, CrWB and CrNiW appear. Obviously, in the regimes with a gradual increase in pulse energy, a reduction in WC → W_2_C → WC_1−x_ is initiated and a decrease until the full reduction of W is initiated.

According to the X-ray phase analysis, the predominant phases are intermetallic compounds and solid solutions with iron from the substrate, and the main wear-resistant phases are TiB_2_, TiB, W_2_C and WC_1−x_. In addition, small amounts and traces of B_4_C, TiB, WB, Fe_2_B, FeB, Cr_3_B_4_, CrB_4_, Ni_3_B, W_2_CoB_2_, Ti_4_N_3_B_2_, CoW_3_C_4_, Fe_0.7_N_0.3_ and TiN_0.3_ were found, the amounts of which increase with increasing pulse energy. The amount of TiB_2_ and B_4_C is much less than in the hard alloy electrode material, and WC is almost not detected, which indicates its dissociation (or transformation). In the composition of most of the obtained coatings, instead of B_4_C, new iron, chromium and tungsten borides are observed, as well as ternary ones of the W_2_CoB_2_ type.

The registered new phases, although in low concentration, are distinguished by high hardness, wear resistance, corrosion resistance and chemical stability in aggressive environments. The X-ray data show that the phase composition of carbon 45 steel is enriched with more carbides and borides than in ESD with conventional hard alloy electrodes, which suggests a significant improvement in the operational properties of the coated surfaces.

The comparison of the phase composition of the coatings from the studied electrodes shows that during ESD with the KWW10B10T20 electrode at a pulse energy of 0.05–0.07 J, the number and complex amount of carbide, nitride, boride and intermetallic phases are higher than those of the coatings obtained with the other electrodes.

**Figure 11:**
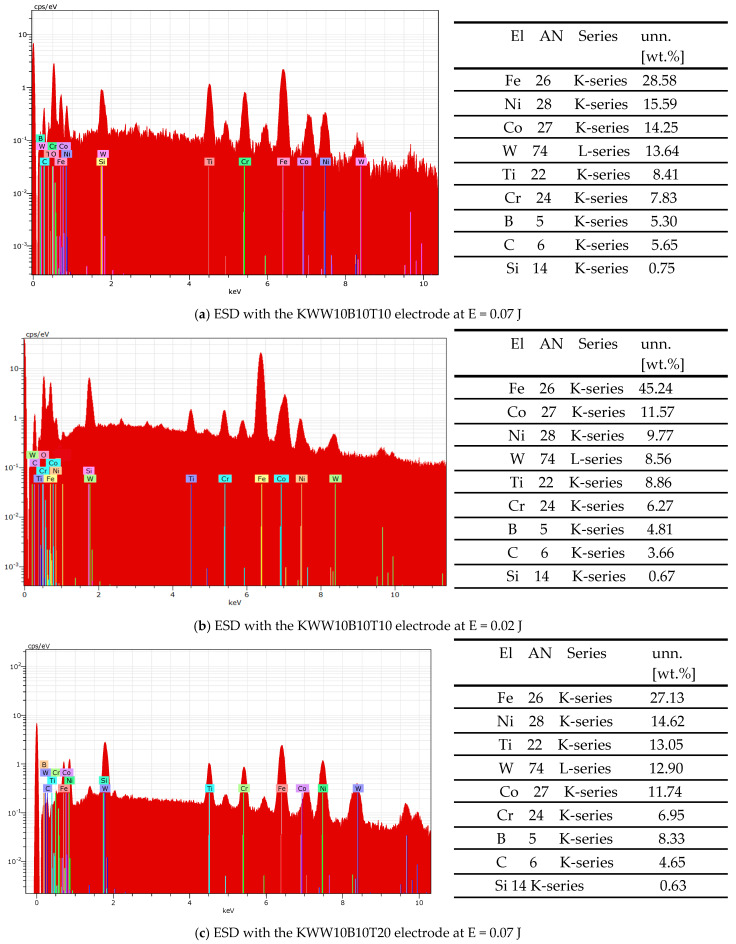
Energy-Dispersive Spectroscopy (EDS spectrum) of coatings from the KWW10B10T10, KWW10B10T20 and KWW10B10T20 electrodes.

The data obtained from the X-ray structural analysis for the phase composition of the coatings are consistent and were confirmed by the data from the SEM analyses of the topography, structure and morphology (Figure 6 and Figure 7) and from the Energy-Dispersive Spectroscopy (EDS) analyses of the elemental composition of the coatings (Figure 11).

The EDS analysis shows that all elements from the electrodes and the substrate are found in the composition of the coatings. In all coatings, the presence of iron from the substrate is predominant (Figure 11). With increasing pulse energy, the amount of elements from the electrode materials also increases at the expense of a decrease in the amount of iron (Figure 11a,b). Small amounts of oxygen are found in the coatings, which is an indicator of oxidation in the process of spark discharges. As can be expected, the maximum amount of elements from the solder mass is registered in the coatings of the KWB10T10 and KWW10B10T10 electrodes, and the maximum amount of high-hardness and wear-resistant compounds is found in the coatings of the KWW10B10T20 and KWW10B20T20 electrodes at a pulse energy of E ≥ 0.05 J. Based on the results, it is established that titanium and tungsten practically do not diffuse into the base, while iron from the base actively diffuses into the coating, which can be explained by the fact that titanium and tungsten carbides and borides are more difficult to melt and crystallize earlier than iron.

The instantaneous melting of the metals from the electrode solder mass and the carbide and boride grains, followed by their super-rapid cooling, led to the formation of a fine-grained ultradispersed structure with the crystallite size of the phases registered in the composition of the coatings in the range of 10–80 nm, which suggests that nanoscale structures are formed in the coatings. The presence of nanoscale structures in ESD has been reported in the works of numerous researchers [1,5,6,8,15,16,17,27,31]. Along with the above, the broadening of the diffraction peaks of Fe, metal alloys and intermetallic phases reflects the formation of both solid solutions and new compounds in the resulting anode–cathode mixture, as well as the refinement of the structure, which ultimately reaches a structureless state. The presence of “glass-like” zones (Figure 6 and Figure 7) also suggests the presence of a certain partial “amorphization” of the coating.

If we take into account the influence of low pulse energy and multicomponent electrodes with low-melting binding metal mass used in ESD, as well as extremely high heating rates, namely 10^4^ °C/s with extremely fast supercooling and the solidification of the molten anode–cathode mixture according to literature data, then with a high degree of probability, it can be assumed that coatings with a fine, superdispersed crystalline and amorphous–nanocrystalline structure are formed on the cathode. The comparison of the SEM images and X-ray structural models of the studied coatings (Figure 6a–e and Figure 7a,b,e,f) shows that the coatings of KWB10T10 and KWW10B10T10 are more uniform and smoother and form without protrusions and irregularities, with glass-like zones prevailing. The surface of the coating of the KW10B10T20 electrode is quite diverse in terms of its structure. A mixture of amorphous and crystalline phases is observed (as established by the XRD analysis). In the coatings of KWB20T20 and KWW10B20T20 (Figure 7a–e), more irregularities and a smaller amount of glass-like areas are visible. SEM analysis (Figure 6 and Figure 7) and the analysis of the X-ray diffraction patterns show that the largest amount of glass-like areas is registered in the coatings from the electrodes with the highest amount of the binding metal mass, namely KWT10B10 and KWW10T10B10 at a pulse energy of E = 0.03–0.07 J. Apparently, this pulse energy creates smaller sizes of the molten cathode spot; therefore, a sufficiently high cooling rate of the mixed melt is achieved, and coatings with an increased amount of amorphous phases, with reduced roughness, increased their density and uniformity.

With a further increase in energy to 0.3 J, the size and volume of the cathode spot increase, and the cooling rate of the melt decreases, and accordingly, the amount of glass-like amorphous–nanocrystalline zones decreases. The presence of amorphous phases in the composition of the coatings has been reported by many authors [5,6,7,8,9,10,11,12,13,14,16,19,27,39], but most of them use mainly metal alloys or nano-sized alloys with “amorphous-forming” components as electrodes, and the production of amorphous structures from conventional hard alloy electrodes has been reported only by individual researchers [10,15,17]. Glass-like zones are obtained in the coatings from all electrodes experimented on in this work, and their highest amounts are obtained at pulse energies in the range of 0.03–0.07 J. As a result of the lower melting point, the NiCrBSiCFe alloy is used as a precursor in the composition of the electrodes containing amorphizing elements (such as B, Si and C), and the low pulse energy during ESD and the degree of amorphization of the resulting coatings are increased compared to coatings made from conventional WK8 hard alloys.

Amorphous and nanoscale surfaces can be formed using various ways, such as CVD and PVD methods, electron beam and laser surface treatments, hot isostatic pressing, self-propagating high-temperature synthesis, etc., but they require expensive equipment, large investments, complex technologies and high costs, which are not always economically feasible and justified. Some authors report on obtaining amorphous and nanostructured surfaces with lower-cost methods such as plasma detonation, high-velocity thermal spraying (HVOF), plasma spraying, etc. Koga et al. [39] obtained an amorphous surface via thermal spraying with Fe-Cr-Nb-B alloys. Metal–glass coatings have also been obtained using sol–gel technology. Myasoedova et al. [51] reported on the creation of layers of amorphous gels, metal and ceramic nanomaterials. The sol–gel process has been proposed as a cheaper and lower-temperature alternative to traditional methods such as sputtering, CVD and plasma spraying for the deposition of thin ceramic coatings. Due to its simplicity, low cost and flexibility; easy technology; the possibility of local application of the coatings; strong metallurgical bonds; and the high wear resistance of the coatings, the ESD process has emerged as the technically and economically most advantageous alternative to the above methods for creating amorphous and nanoscale surfaces. Many authors claim that ESD is the lightest and easiest method for obtaining amorphous surfaces [6,7,8,9,10,11,12,13,14,16,17,18,19,27,31].

The microhardness of the coatings is 3–5 times higher than that of the steel substrates (Table 5). Due to the presence of many components with different hardness and the uneven distribution of the high-hardness phases in the layer, the individual measured values of the microhardness HV of the applied coatings vary in a very wide range of −8–20.6 GPa, which does not allow us to establish a clear dependence and correct assessment of the influence of the composition of the electrode and the pulse energy, despite the tendency to increase the microhardness with increasing pulse energy (Table 4). Separate measured peak values of microhardness up to 19–20 GPa are observed at a metal matrix content of 22–27% in the coatings from the KWW10 B20T20 and KWW10B10T20 electrodes, the composition of which is most enriched in high-hardness phases and intermetallic compounds. Due to the lower content of WC, B_4_C and TiB_2_ in the coatings from the KWW10B10T10 electrode, the measured values are slightly lower but are close to each other, as they are predetermined by their almost identical phase composition and thickness.

The microhardness values presented in Table 4 exceed those obtained with conventional carbide electrodes in the works of [6,7,8,9,10,11,12,13,14,16,17,18,19,27,31], as well as slightly exceed those of coatings from similar multicomponent electrodes [35,45,48], but are much lower than those presented in [30], where B_4_C-TiB_2_-sintered alloys without a binding metal mass were used as electrodes, and values of 16–40 GPa were reported.

The obtained data allow us to determine the range of pulse energy, from 0.03 to 0.07 J, in which the amount of high-hardness compounds from the electrode material and the formed new carbides, borides, intermetallic compounds and amorphous–crystalline structures and the geometric characteristics of the coatings are most comprehensively combined. Within the determined range of pulse energy, the most favorable from the point of view of the composition of the coatings is the KWW10B10T20 electrode, and in terms of the geometric characteristics and thickness of the coatings, the KWW10B10T10 electrode is the most favorable.

### 3.3. Tribological Tests

From the conducted studies of the change in the coefficient of friction (µ), the tangential force Ft, the traces of the “scratch” tests and the acoustic emission as a function of the normal load Fn, it was found that the coefficient of friction, the tangential force, the acoustic emission and the dimensions of the traces of the scratch tests for all the studied coatings have close values that increase monotonically with the increase in the normal load. However, with an increase in the energy of the pulses, due to the higher roughness of the obtained coatings, the coefficient of friction µ, the tangential force Ft and the acoustic emission take on higher values, but the differences are relatively small despite the differences in their roughness parameters; e.g., for coatings from the electrode KW10B10T20 at E = 0.16 J with a normal load of Fn = 50 N, the coefficient of friction is µ ≈ 0.33, and at E ≈ 0.03, it is J—µ ≈ 0.28. The coefficient of friction values of the coatings from the studied electrodes at a pulse energy of E = 0.03–0.3 J are close both to each other and to those of the substrate, and at Fn ≈ 50 N, they vary within the range of µ = 0.26–0.35.

**Figure 12:**
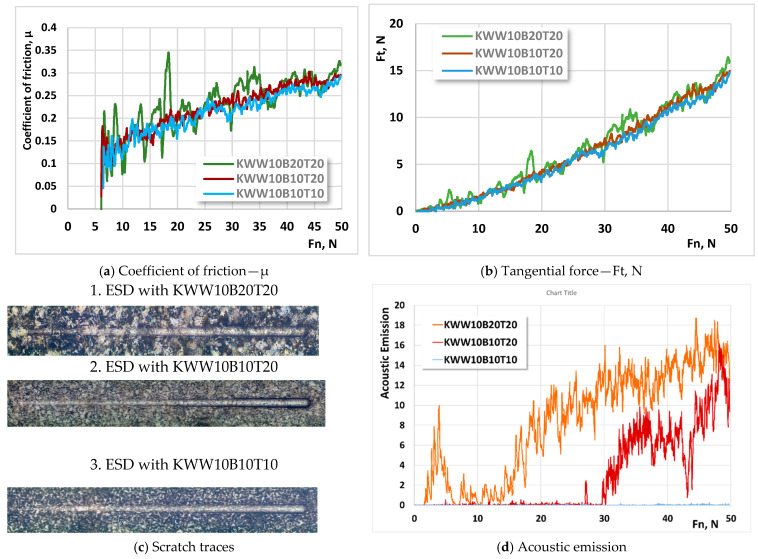
COF, tangential force, scratch traces and acoustic emission of ESD coatings at E = 0.07 J versus the normal force.

The fluctuations of µ in the coatings obtained with higher energy, however, are noticeably higher, most likely due to the higher roughness of the coatings. The analysis of the obtained values reveals that from the point of view of the change in µ and Ft, the most suitable regimes are those with an energy of 0.03–0.07 J. The above findings are confirmed by the data presented in Figure 12, which illustrates the change in the coefficient of friction, the tangential force Ft, the traces of the “scratch” tests and the acoustic emission as a function of the normal load Fn of the coatings from electrodes KWW10B20T20, KWW10B10T20 and KWW10B10T10 that are deposited at a pulse energy of E = 0.07 J. The figure shows that the lowest values of the coefficient of friction, Ft and scratch test traces are shown by the coatings from KWW10B10T10. The higher values of µ, Ft and the sizes of the scratch test traces, as well as the fluctuations in their values in the coatings from the KWW10B20T20 electrode, are clearly due to the higher roughness and unevenness of the surface of these coatings and probably also due to the higher content of solid phases in the coating.

The values of the coefficient of friction, tangential force, acoustic emission and scratch test traces are 10–15% lower than those of coatings from conventional hard alloy electrodes presented in the works of [22,29,32,34], which allows us to assume that coatings from “KWW” electrodes have higher plasticity and toughness and better adhesion to the deposited surface than those obtained with tungsten-free and conventional tungsten hard alloys, making them more suitable for applications with impact loads. Normal loading has a relatively mild effect on the friction coefficient, which indicates that friction occurs under conditions of predominantly elastic contact, which is more pronounced in coatings from the KWB10T10 and KWW10B10T10 electrodes. This is also evidenced by the absence of acoustic emission, which is obviously due to the higher content of the metal bond in the coatings. The coatings of the KWB10T10 and KWW10B10T10 electrodes applied at pulse energies up to 0.07 J do not show a loss of cohesive strength at a load of up to 50 N, which is an indicator of very good plasticity but also lower hardness. The absence of acoustic emission in these coatings (Figure 12d) is an indicator of their higher toughness and strength, which are determined by the higher content of the metal-binding mass. The acoustic emission of the coatings from KWW10B10T20 is also absent and occurs at normal loads of Fn ≥ 30 N, but its values remain lower than those of the coatings from the KWW10B20T20 electrode. In the coatings of the KWW10B20T20 electrode (Figure 12c), fine cohesive cracks are observed in the trace of the analysis at the beginning of the application of the load, the size and quantity of which increase with increasing load. This indicates higher brittleness, and the higher value of the acoustic signal is evidence of the higher hardness of the surface of these coatings.

**Figure 13:**
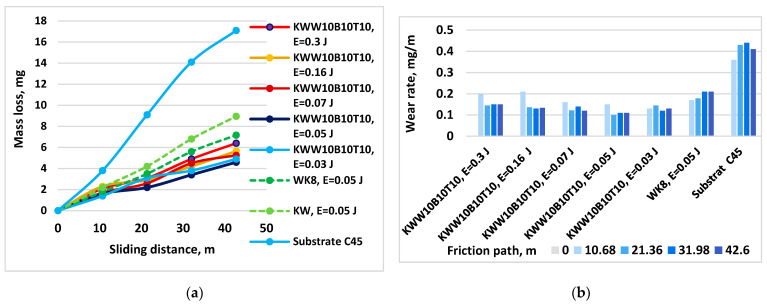
Mass loss (**a**) and wear rate (**b**) of coatings from the KWW10B10T10 electrode as a function of the friction path and pulse energy at a load of 10 N.

Figure 13 shows the mass loss and wear rate of coatings from the KWW10B10T10 electrode as a function of the friction path and pulse energy at a load of 10 N.

**Figure 14:**
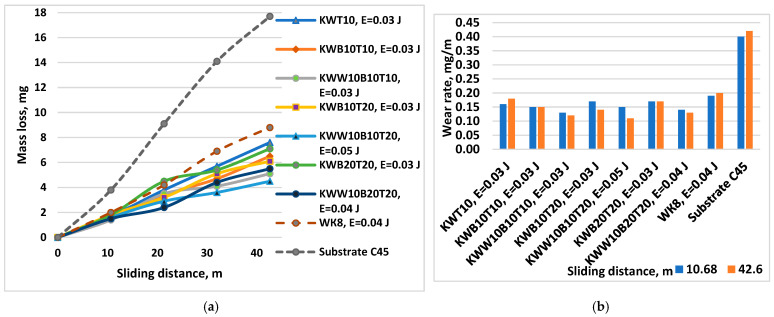
Mass loss (**a**) and wear rate (**b**) of coatings from the “KW” and “KWW” electrodes as a function of the friction path and pulse energy at a load of 10 N.

Figure 14 shows the wear as the mass loss and wear rate of coatings applied at an energy of 0.03–0.07 J with electrodes of the “KW” and ”KWW” types versus the friction path at a load of 10 N. The coatings were applied with the maximum possible energies at which the maximum thickness at a roughness of Ra = 3–4 µm was recorded for the respective electrodes.

**Figure 15:**
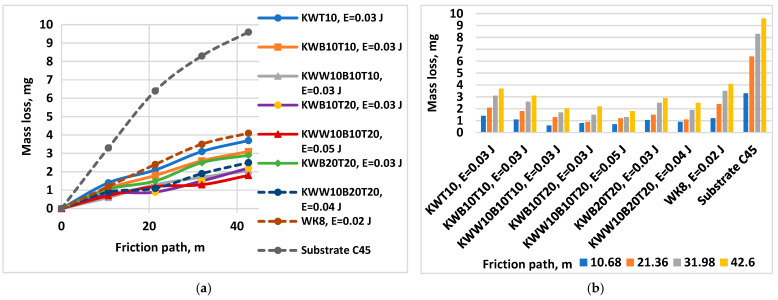
Mass loss (**a**) and wear rate (**b**)of coatings from the KW and KWW electrodes as a function of the friction path and pulse energy at a load of 5 N.

Figure 15 shows the wear (a) as the mass loss and wear rate (b) of coatings applied at an energy of 0.03 J with electrodes of the “KW” and “KWW” types versus the friction path at a load of 5 N.

The results obtained show that the wear of all coated samples is 3–5.5 times lower than that of uncoated C45 steel. The changes in the mass wear, wear rate and wear resistance of the coatings are similar. The wear curves of the coated samples (Figure 13a, Figure 14a and Figure 15a) at both loads are located in a narrow range and are significantly distant from that of the uncoated steel. In a similar narrow range are also the values of the standard deviation of the coated samples, which are lower than those of the uncoated sample. The standard deviation of the wear values is different for the samples coated with the different electrodes and pulse energy studied, as well as for the different friction path traveled, which varies within 6–14%. The differences in the deviation at the different electrode compositions and the same pulse energy, however, are relatively small, which is due to the close composition of the electrodes and the same effect of the pulse discharges. With the increase in the friction path, the deviation values monotonically increase. The highest values of the standard deviation up to 14% were recorded for samples coated with the KWW10B20T20 electrode in the modes with pulse energy values of E ≥ 0.05 J, which is apparently due to the higher roughness and unevenness of the coatings at these pulse energy values, and the lowest values were recorded for samples coated with the KWW10B10T10 and KWW10B10T20 electrodes at pulse energy values of 0.03 and 0.05 J and friction paths of 11 and 21 m.

The transferred WC, TiB_2_ and B_4_C particles trapped in the metal matrix of the coating increase its wear resistance, and on the other hand, the formation of new borides, carbides, intermetallic and ultrafine structures further contributes to the increase in wear resistance. While the influence of roughness parameters on wear can be distinguished and taken into account in mechanical processing, in ESD coatings, there is a simultaneous complex influence of both roughness and the changed composition, structure and properties of the coatings, and the distinction of these two sides is complicated. The results show that the classical surface microgeometric indicators (Ra, Rz and Rt) in this case do not reflect the actual state of the frictional contact.

With increasing pulse energy values up to 0.05 J, regardless of the increasing roughness, the coatings demonstrate higher wear resistance than those applied at both lower energy and lower roughness, followed by that of the substrate. This is due to the fine-grained structure, high hardness and wear resistance of the coating materials. In addition, the plasticizing metal matrix acts as a lubricant during friction, thereby preventing the oxidation of the steel surface, the formation of wear craters and the welding and seizing of the rubbing surfaces. Observations of the wear traces showed that the wear mechanism of the studied ESD coatings is similar under both loads, and the predominant types of wear are abrasive and are caused by the hard particles of the sandpaper and adhesive.

The wear of the coated surfaces begins from the unmelted particles transferred by the brittle fracture of the electrode, which are not firmly connected to the base and have the highest peaks of micro-roughness, which, under the action of the abrasive particles, are broken off and separated from the coating. The separated solid particles get stuck between the rubbing surfaces, accelerating the development of wear. However, due to their higher hardness and good plasticity, the coatings slow down the development of wear over time, providing increased wear resistance of the coated surfaces. Based on the results obtained and our previous studies [30,36,38,43,48], the following generalizations can be made:

- The influence of pulse energy on wear can be clearly seen in Figure 13. With increasing energy, a tendency towards a decrease in the wear of KWW10B10T10 coatings is observed. At E = 0.05 J, wear reaches a minimum and then gradually increases. From Figure 13b, it is easy to determine the energy range in which the wear rate is minimal, E ≈ 0.03–0.16 J, with the least wear being recorded at E = 0.05 and 0.07 J. Within this range, the differences in the wear of the coated samples are relatively small (Figure 13a). From the metallographic, SEM and EDS analyses, it was established that in ESD with the KWW10B10T10 electrode at pulse energies of 0.03–0.07 J, the coatings achieve an optimal and most favorable balance in terms of wear resistance between, on the one hand, the roughness, uniformity and thickness of the coating, and, on the other hand, the amount of solid phases transferred to the cathode, newly formed wear-resistant phases and amorphous-crystalline structures, which play a decisive role in reducing wear. Apparently, at lower and medium energies up to 0.07 J, wear is mainly influenced by the content of wear-resistant phases and the higher microhardness and changes in the structure of the surface layer; the negative influence of increasing roughness with increasing energy in the direction of 0.03 to 0.07 J is compensated by the thickness, composition and structure of the coatings. With a further increase in the pulse energy above 0.07 J, the amount of material transferred from the brittle fracture in the solid phase increases, and the roughness, unevenness and structural defects of the coatings also increase (Table 4 and Table 5 and Figure 6 and Figure 7), and the amount of amorphous structures decreases, which causes a gradual increase in wear. Due to the fine-grained amorphous–crystalline structure and the presence of greater thickness and a large amount of phases with high hardness and wear resistance, at E = 0.07 and 0.16 J, the increase is very slight, with the wear values remaining comparable to those at E = 0.05 J, and even at E = 0.3 J, its values remain up to 2.5–3 times lower than those of uncoated steel but higher than those at E = 0.03–0.07 J. In the coatings from the other electrodes studied, the same tendency towards a decrease in wear is observed until certain values of pulse energy are reached, which are different for each specific electrode, after which an increasing trend is registered at E = 0.05 J. For the different electrodes with a further increase in energy, the effect of ESD decreases to 2–2.5 times lower wear than that of the substrate. Apparently, the roughness and unevenness of these coatings, which are significantly higher, gradually acquire a predominant influence on wear, which leads to its increase. This gives us a reason to recommend the use of pulse energy in the range of 0.03–0.07 J as a more favorable alternative for ESD with KWW10B10T10;
- Figure 14 shows the wear of the coatings applied with the studied electrodes at the “limit” pulse energy, with a further increase resulting in an increase in wear. The data from the figure show that the values of the limit pulse energy increase with increasing amounts of the binding metal mass in the electrode. However, for coatings from the KWT10, KWB10T10 and KWW10B10T10 electrodes, it can be assumed that the energy range at which the wear is lowest is E = 0.03–0.16 J (Figure 13a,b and Figure 14), and the range of the lowest wear of coatings from KWW10B10T20 is E = 0.03–0.07 J, and for that from electrode KWW10B20T20, the range is 0.03–0.05 J. As established above, the presence of higher amounts of the refractory high-hardness WC, B_4_C and TiB_2_ at the expense of the binding metal mass increases the hardness but also the brittleness by weakening the strength characteristics of the electrode, and as a result, the transfer of brittle fracture increases at lower pulse energies;
- The analysis of the influence of the added 10% WC-Co shows that the coatings of the “KWW” electrodes demonstrate lower wear than that of the ESD with the “KW” electrodes. The comparison of the wear and wear rate (Figure 15) shows that the samples coated with KWW10T10B20 and KWW10T10B10 have at the very least wear at both load values of 5 and 10 N, up to 1.2 to 1.7 times lower than that of the samples coated with the other electrode compositions and more than 2 times lower than that of the samples coated with the classic hard alloy WK8. These results are consistent with the conclusions reached in the works of [15,29,31,32,35,44], where WC and NiCrBSi self-fluxing alloys were used for wear-resistant coatings. However, from the wear data (Figure 13, Figure 14 and Figure 15), it can be established that the differences in the wear of the samples coated with KWW10T10B20, KWW10T10B10 and KWW10T20B20 are relatively small. It is logical to expect that the increased content of B_4_C and TiB_2_ to 20% will demonstrate the lowest wear. However, in the ESD process, some of the transferred incompletely melted electrode particles of KWW10T20B20 do not adhere to the substrate, and those that do adhere do not fully embed and do not adhere well to the molten metal matrix (Figure 7a,c,d), worsening the roughness, uniformity, adhesion and structural characteristics of the coating. As a result, the wear of the KWW10T20B20-coated electrode increases by ≈10–30% compared to the minimum obtained in ESD with KWW10T10B20 but nevertheless remains up to 2.5–3.5 times lower than that of the uncoated substrate (Figure 14a and Figure 15a). The change in the wear rate is similar. Apparently, a higher concentration of high-hardness materials leads to a weakening of the bonds of the individual grains with the metal matrix in the electrode and in the coating, thereby resulting in an increase in wear; moreover, the broken-off high-hardness particles in the friction process act abrasively on the surface of the coating, contributing to its further increase.

Based on the results obtained in this work, for each of the electrodes used, the limit values of the pulse energy can be determined with sufficient accuracy, after which wear begins to decrease. Since the values of the universal roughness parameter Ra for each specific electrode to some extent correspond to the values of the pulse energy, based on the results obtained, it can be assumed with approximation that for coatings with values of Ra >≈ 3.5 µm, the wear resistance shows a tendency to decrease.

The summary of the obtained results shows that the use of multicomponent electrodes for ESD with the composition Co-Ni-Cr-B-Si-Fe-C-WC-B_4_C-TiB_2_ allows for the creation of coatings from a predominantly liquid phase with better uniformity, lower roughness and porosity and higher density, thickness, microhardness and wear resistance compared to coatings obtained with conventional hard alloy and multicomponent electrodes known in the literature. In the ESD process, a larger number of newly formed wear-resistant phases and intermetallic compounds were synthesized in the coatings, and an increased amount of amorphous–nanocrystalline structures improved adhesion to the steel base, resulting in lower wear of the coated surfaces compared to those coated with the conventional hard alloy electrodes. The largest amount of amorphous–nanocrystalline regions is registered in the coatings from the electrodes with the largest amount of binding metals, namely KWT10B10 and KWW10T10B10 at pulse energy values of E = 0.03–0.07 J. It is widely known that amorphous metals, compared to the crystalline state, have improved properties such as higher strength, hardness and corrosion resistance. There is currently no sufficient data in the literature on the amorphous structures from the multicomponent hard alloys obtained in this work. Although numerous authors declare the production of surfaces with amorphous and nanocrystalline structures using ESD, the properties of these structures and their influence on the wear resistance of coated surfaces have not yet been sufficiently studied, and their study raises the need for further research.

The use of electrodes with a higher content of binding metals (30–40%) allows for the production of coatings with increased thickness at lower roughness. The electrodes KWT10, KWB10T10 and KWW10B10T10 allow for the use of higher pulse energies and the production of more uniform and homogeneous coatings with better geometric characteristics and a structure with predominantly glass-like zones. The maximum thickness at which relatively uniform coatings with roughness up to Ra = 3–4 µm are obtained using ESD at E= 0.07 J is on the order of 45–50 µm. In the determined most suitable pulse energy range of 0.03–0.07 J, the electrode KWW10B10T20 emerges as the most favorable in terms of the composition and wear resistance of the coatings, and in terms of the geometric characteristics and thickness of the coatings, the electrode KWW10B10T10 is the most favorable.

Based on the results of the above studies, it can be stated that the use of the “KWW” electrodes of WK8, TiB_2_ and B_4_C with a metal-binding mass for Ni-Co-Cr-B-Si-Fe-C-WC and the ESD method with low pulse energy can allow us to obtain coatings with improved topography and morphology and results in a significant improvement of the properties of steel surfaces, such as microhardness, COF, friction force, adhesion and wear resistance, compared to those of ESD with conventional WC-Co electrodes, and optimal results have been obtained.

## 4. Conclusions

By using ESD with multicomponent electrodes containing WC, Co-Ni-Cr-B-Si-Fe-C semi-self-fluxing alloys and additives of the superhard compounds B_4_C and TiB_2_, as well as using low-energy pulses, dense and uniform coatings with improved adhesion to the substrate, crystalline–amorphous structures and thickness, roughness and microhardness that can be changed by the ESD modes in the ranges of δ = 15–70 µm, Ra = 1.5–7 µm and HV = 8.5–15.0 GPa, respectively, as well as improved physicochemical and tribological properties, were obtained.

The microhardness of the coatings is 3–5 times higher than that of the steel substrates, as the differences in the microhardness values of the coatings from different electrodes vary within the range of 1–3.5 GPa. The highest microhardness is that of ESD with the KWW10B20T20 and KWW10B10T20 electrodes, whose composition is most enriched in high-hardness phases.

In addition to the phases from the deposition electrode, new highly resistant phases and compounds were also registered in the coatings, the amounts of which increase with increasing energy.

The presence of nanocrystalline–amorphous structures was registered in the coatings, whereby the largest amount of which was recorded in ESD with electrodes KWT10B10, KWW10T10B10 and KWW10B10T20 at pulse energies of E = 0.03–0.07 J. The ranges of pulse energy at which the amount of nanocrystalline–amorphous structures regions is maximum have been determined for each electrode.

The values of coefficient of friction (COF), tangential force, acoustic emission and scratch marks of coatings from the “KW” and “KWW” electrodes are 10–15% lower than those of coatings from conventional carbide electrodes. The lowest values of COF, Ft and scratch marks are shown by coatings from the KWB10T10 and KWW10B10T10 electrodes, which were applied at pulse energies of up to 0.07 J. They do not show a loss of cohesive strength at loads of up to 50 N, which is an indicator of very good plasticity but lower hardness. The highest values are shown by coatings from the KWW10B20T20 electrode, and the higher value of the acoustic signal indicates the higher brittleness of these coatings.

With increasing energy, the wear of the coatings decreases, reaching a minimum in the energy range of 0.03–0.07 J. The samples coated with KWW10T10B20 and KWW10T10B10 showed the lowest wear at both load values of 5 and 10 N. For each of the electrode compositions studied, the energy range at which the wear of the coatings is minimal and the limit values of the pulse energy have been determined, after which the wear begins to increase.

The amount of the binding metal mass from 20 to 40%, depending on the energy used, affects to a varying extent the parameters and characteristics of the obtained coatings. Increasing the amount of the binding metal mass to 32–40% (KW10B10T10 and KWT10) expands the range of possible modes in which dense and uniform coatings are obtained, contributes to better adhesion of the coating to the base and allows for the use of higher pulse energies (up to E = 0.16 J); the production of coatings with increased thickness, strength and toughness; and a high amount of amorphous-like phases with acceptable roughness (Ra ≈ 4–5 µm), but lowers microhardness and wear resistance under friction.

The specified ratios between the individual components in the composition of the electrode material and the ESD conditions allow for the maximum improvement of the properties and wear resistance of the coatings, up to 4–5 times higher than that of the substrate and up to 1.5 times higher than that of conventionally used electrodes.

The results of the present research confirm the positive effect of coatings from alloys of the Co-Cr-Ni-Si-B-C system with additions of WC and TiB_2_ B_4_C on the structure and performance characteristics of C45 steel.

## Figures and Tables

**Table 1:** Chemical composition of the bonding powder mixtures.

Element, wt.%	C	Si	Cr	Fe	B	Co	Ni	Designation of Composition
CoNiCrBSi	1.5	1.5	23	0.5	1.5	40	balance	KW = 45%CoNiCrBSi + 55%WC

**Table 2:** Composition and designation of multicomponent electrode materials for ESD.

Designation/Composition, wt.%	KW	WC-Co8	TiB_2_	B_4_C
KWT10	90	-	10	-
KWB10T10	80	-	10	10
KWW10B10T10	70	10	10	10
KWB10T20	70	-	20	10
KWW10B10T20	50	10	20	20
KWB20T20	60	-	20	20
KWW10B20T20	50	10	20	20
WK8		92/8		

**Table 3:** Used ESD modes with the vibrating electrode.

№ of Regimes	1	2	3	4	5	6
Capacity, C, µF	5	10	15	20	50	100
Pulse energy E, J	0.02	0.03	≈0.05	≈0.07	≈0.16	≈0.3

**Table 4:** Parameters of coatings obtained with ESD with the vibrating apparatus.

№	Electrode, E = 0.03–0.3 J	Ra, μm	δ, μm	Hv, GPa	Coeff. of Hardening
1	WK8	1.8–5.5	14–42	7.8–12	3–5
2	KWT10	2.5–5.3	19–66	8–12	3–5
3	KWB10T10	2.5–5.3	18–63	8.3–12.5	3–5.3
4	KWW10B10T10	2.3–5.6	16–59	8.8–13	3.5–5.6
5	KWB10T20	2.2–5.5	16–56	8.8–13	3.5–5.6
6	KWW10B10T20	2.2–5.9	20–53	9.5–14.5	3.6–6.2
7	KWB20T20	2.1–5.8	18–55	9–14	3.8–6
8	KWW10B20T20	2.1–6.8	18–55	9.5–15	3.8–6.4

**Table 5:** Roughness, thickness and growth of the cathode in ESD with the vibrating apparatus.

Electrode, Impulse Energy, J	Roughness and Thickness δ of the Coating After 3 Electrode Passes						Average Thickness	Cathode Growth, mg/cm^2^
	Roughness, µm				Thickness δ, µm		µm	mg/cm^2^
	Ra	Rz	Rq	Rt	min	max		
Substrate	2.38	9.56	2.7	9.65	-	-	-	-
KWW10B20T20, E = 0.07	4.84	14.12	5.50	17.63	27.6	35.5	30	0.67
Deviation, ±, µm	0.88	2.53	0.82	3.72			+5.5, −2.4	0.065
KWB20T20, E = 0.07	4.46	12.72	5.16	15.58	30.2	38.1	33	0.83
Deviation, ±, µm	0.7	1.92	0.72	1.56			+5.1, −2.8	0.067
KWW10B10T10, E = 0.07	4.29	12.62	4.67	14.63	33.6	41.9	36	1.2
Deviation, ±, µm	0.53	1.22	0.65	1.31			+5.9, −2.4	0.105
KWW10B10T10, E = 0.05	4.04	11.8	4.44	13.48	23.7	30.3	28	0.88
Deviation, ±, µm	0.45	0.85	0.33	0.99			+2.3, −4.3	0.077
KWB10T10, E = 0.07	3.87	12.2	3.6	14.11	35.6	43.5	40	1.43
Deviation, ±, µm	0.49	1.22	0.53	1.43			+3.5, −4.4	0.127
KWB10T10, E = 0.05	3.38	9.56	3.7	11.65	26.5	35.9	32	1.05
Deviation, ±, µm	0.38	1.05	0.45	1.24			+3.9, −5.5	0.091

**Table 6:** Registered phases in the coatings of the KW and KWW electrodes.

Phases in Coatings	2θ Angle; °
Predominant phases	
Fe	44.5; 63; 65.18; 82.35
Cr-Ni-Fe-C	43.3; 50.6; 63; 74.06
Cr-Ni-Fe	44.45; 64.84
WC_1−x_	36.9; 43.08; 62.1; 64.85; 74.22; 78.34; 81.8
TiB_2_	27.8; 34.1; 44.4; 61.16;68.1
FeNiCo	44.6; 52.4; 64.5; 74.5
In small quantities and traces	
B_4_C	22; 31.9; 34.9; 41.9; 50.6; 71.89; 88.6
Co	41.4; 44.2; 45.34; 47.3; 76.1
FeNi	44.1; 52.5; 64.5; 74.7; 85; 90.6
Sintered new phases In small quantities and traces	
TiB	36.65; 37.05; 42.95; 61.95; 73.7; 78.7;
WB	21.25;29.9; 36.2; 42.24; 60.12;
FeSi	27.5; 44.75; 49; 57.1; 65.1;
Fe_1.88_C_0.14_	43.4; 45; 60.7; 65.65; 81.88;
FeCr	31.3; 44.8; 55.5; 65.25; 74.9;
Fe_2_B	28.53; 29.86; 45.03
FeB	37.7; 41.2
FeNi_3_	38.15; 44.12; 51.4
Fe_0.7_N_0.3_	45.1; 65; 81.88
Fe_3_W_3_C	42.4; 46.5; 52.3; 59.5; 72.3
W_2_CoB_2_	43.2; 74.1
Cr_3_B_4_	23.55; 30.5; 36.87; 41.7; 46.1; 50.8; 62.8
Co_3_Ti	35.45; 43.55; 50.8; 63.2; 74.7
TiN_0.3_	34.86; 37.65; 39.85; 42.78; 52.22; 62.5; 75.34
Ti_4_N_3_B_2_	37.05; 42.95; 62.25; 74.55; 78.5
Ti_2_O	35.65; 37.05; 38.25; 40.6; 63.8; 70.50; 78.5
Ti_6_O	34.95; 38; 39.93; 52.5; 69.9
Fe_2_O_3_	33.3; 35.7; 49.6

## Data Availability

The original contributions presented in the study are included in the article, further inquiries can be directed to the corresponding author.
